# Modifiable lifestyle factors and lifetime risk of atrial fibrillation: longitudinal data from the Korea NHIS-HealS and UK Biobank cohorts

**DOI:** 10.1186/s12916-024-03400-4

**Published:** 2024-05-13

**Authors:** Hanjin Park, Daehoon Kim, Eunsun Jang, Hee Tae Yu, Tae-Hoon Kim, Dong-min Kim, Jung-Hoon Sung, Hui-Nam Pak, Moon-Hyoung Lee, Gregory Y. H. Lip, Pil-Sung Yang, Boyoung Joung

**Affiliations:** 1 Division of Cardiology, Department of Internal Medicine, Yonsei University College of Medicine, 50-1 Yonsei-ro, Seodaemun-gu, Seoul, 03722 Republic of Korea; 2 Division of Cardiology, Department of Internal Medicine, College of Medicine, Dankook University, Cheonan, Republic of Korea; 3 Division of Cardiology, CHA Bundang Medical Center, CHA University, Seongnam, Republic of Korea; 4 Liverpool Centre for Cardiovascular Science at University of Liverpool, Liverpool John Moores University and Liverpool Heart & Chest Hospital, Liverpool, United Kingdom; 5 Department of Clinical Medicine, Aalborg University, Aalborg, Denmark

**Keywords:** Lifetime risk, Atrial fibrillation, White, Asian, Lifestyle factor

## Abstract

**Background:**

The reason for higher incidence of atrial fibrillation (AF) in Europe compared with East Asia is unclear. We aimed to investigate the association between modifiable lifestyle factors and lifetime risk of AF in Europe and East Asia, along with race/ethnic similarities and disparities.

**Methods:**

1:1 propensity score matched pairs of 242,763 East Asians and 242,763 White Europeans without AF were analyzed. Modifiable lifestyle factors considered were blood pressure, body mass index, cigarette smoking, diabetes, alcohol consumption, and physical activity, categorized as non-adverse or adverse levels. Lifetime risk of AF was estimated from the index age of 45 years to the attained age of 85 years, accounting for the competing risk of death.

**Results:**

The overall lifetime risk of AF was higher in White Europeans than East Asians (20.9% vs 15.4%, *p* < 0.001). The lifetime risk of AF was similar between the two races in individuals with non-adverse lifestyle factor profiles (13.4% vs 12.9%, *p* = 0.575), whereas it was higher in White Europeans with adverse lifestyle factor profiles (22.1% vs 15.8%, *p* < 0.001). The difference in the lifetime risk of AF between the two races increased as the burden of adverse lifestyle factors worsened (1 adverse lifestyle factor; 4.3% to ≥ 3 adverse lifestyle factors; 11.2%). Compared with East Asians, the relative risk of AF in White Europeans was 23% and 62% higher for one (hazard ratio [HR] 1.23, 95% confidence interval [CI] 1.16–1.29) and ≥ 3 adverse lifestyle factors (HR 1.62, 95% CI 1.51–1.75), respectively.

**Conclusions:**

The overall higher lifetime risk of AF in White Europeans compared with East Asians might be attributable to adverse lifestyle factors. Adherence to healthy lifestyle factors was associated with the lifetime risk of AF of about 1 in 8 regardless of race/ethnicity.

**Supplementary Information:**

The online version contains supplementary material available at 10.1186/s12916-024-03400-4.

## Background

Atrial fibrillation (AF), the most common sustained cardiac arrhythmia, is a burgeoning global health burden that is associated with substantial morbidity, mortality, and health care costs [1–6]. The prevalence of AF is expected to rise to about 15 million by 2050 in the US, 17.9 million by 2060 in the European Union, and 9 million by 2050 in China [7, 8]. Therefore, preventive and intervention strategies for AF are a public health priority and several short-term risk factors for incident AF have been identified [9–12]. Many of those AF drivers are potentially reversible and occur in multiple combinations that may additively contribute to incident AF [9]. Thus, understanding the association between multiple combinations of adverse lifestyle factors and the risk of AF is important in designing preventive strategies for incident AF.

Lifetime risk is a useful quantification of the absolute risk over a person’s lifetime and is vital from the public health perspectives that complement short term risk prediction. The lifetime risk of AF has previously been reported in Whites [13–18] and Asians [19, 20]. However, the reason for the higher incidence of AF among Europeans compared with Asians are yet to be fully understood [21]. This is important given that East Asia represents 21.5% of the global population and is the fastest growing minority group in Western countries [22].

In this study, we compared the lifetime risk of AF according to modifiable lifestyle factors in an East Asian cohort and assessed the differences with that from White Europeans. Our estimates might provide a more precise picture of the current and future public health implications of AF and its association with modifiable lifestyle factors, along with similarities and disparities between Asian and Western people who develop AF. We used longitudinal population-based cohort study data from the Korea National Health Insurance Service-Health Screening cohort (K-NHIS-HealS) and UK Biobank.

## Methods

### K-NHIS-HealS cohort

The K-NHIS-HealS cohort is a population-based, retrospective cohort study comprising 514,764 adults recruited using the 10% random sampling method from a total of 5.5 million adults in the National Health Information Database [23, 24], which is based on the national health claims database established by the National Health Insurance Service (NHIS) of South Korea. The K-NHIS-HealS cohort contains data from biennial health screening examinations including information on sociodemographic and anthropometric variables, laboratory measurements, health care utilization, and health outcomes [25]. Among the 514,764 participants in the K-NHIS-HealS, we included 425,610 individuals aged 45 to 84 years who participated in health screening examination between January 1, 2005 and December 31, 2010. We excluded participants with prevalent AF (*n* = 4924) and missing modifiable lifestyle factor data (*n* = 28,931). A total of 391,755 individuals were eligible for the analysis. All participants in the K-NHIS-HealS were East Asians.

Incident AF was defined as the first of at least two outpatient visits on different days or the first admission or death with the diagnosis of AF. The diagnostic accuracy of incident AF in the K-NHIS-HealS was validated in a previous study to have a positive predictive value of 94.1% [26]. Participants were followed until incident AF, death, censoring, or December 31, 2013, whichever came first (Additional file 1: Fig. S1).

### UK Biobank

UK Biobank is a population-based, prospective cohort of 502,424 individuals recruited between 2006 and 2010. Individuals who agreed to take part in UK Biobank attended one of 22 assessment centers across England, Wales, and Scotland and provided baseline information, medical history, physical measures, life style factors, and biological samples. Further details of the study design and data collection have been described previously [27]. Among the 502,421 individuals in the UK Biobank, we included individuals aged 45 to 84 years who participated in health screening examination between January 1, 2006, and December 31, 2010. We excluded participants with self-reported non-White race/ethnicity (*n* = 24,567), prevalent AF (*n* = 6923), and missing modifiable lifestyle factor data (*n* = 93,459). A total of 331,867 individuals were eligible for the analysis.

Incident AF was extracted from the “first occurrence of health outcomes” field and was obtained by linkage with primary care, hospital inpatient records, and death register. Participants followed until incident AF, death, censoring, or March 31, 2021, whichever came first (Additional file 1: Fig. S1).

Detailed information about the definitions of outcomes and covariates used for K-NHIS-HealS and UK Biobank is provided in Additional file 1: Supplementary Methods, Tables S1 and S2. For an unbiased cross-cohort comparisons, the outcome and covariate definitions were the same for K-NHIS-HealS and UK Biobank.

### Propensity score matching

Among the 391,755 and 331,867 eligible for analysis in the K-NHIS-HealS and UK Biobank, we carried out 1:1 propensity score matching using the nearest neighbor method with a caliper of 0.01. A caliper of 0.01 was chosen because it was 0.2 times the standard deviation of the logit of the propensity score, which has been reported to remove 99% of the bias due to unmeasured confounding [28, 29]. Variables used for propensity score matching were age and sex. After excluding unmatched pairs from both cohorts, 242,763 participants in each of the K-NHIS-HealS and UK Biobank cohorts consisted the final study population (Additional file 1: Fig. S1). We also tested for multiple empirically chosen calipers but found significant difference in age and sex between the two cohorts (Additional file 1: Supplementary Methods).

### Modifiable lifestyle factors

The modifiable lifestyle factors were selected based on the literature [11, 30]. The modifiable lifestyle factors included were blood pressure (BP), body mass index (BMI), cigarette smoking, diabetes, alcohol consumption, and physical activity. We defined non-adverse and adverse categories for each modifiable lifestyle factor as described in Additional file 1: Table S1. Presence of multiple adverse lifestyle factor profiles were evaluated according to two levels: all lifestyle factors were non-adverse (non-adverse) or presence of any adverse lifestyle factor (adverse).

### Statistical analysis

Continuous variables are reported as medians (interquartile range) and compared using the Kruskal-Wallis test. Categorical variables are reported as numbers (percentages) and compared using the chi-square or Fisher’s exact test.

We calculated the lifetime risk of AF from the index age of 45 years to the attained age of 85 years, accounting for the competing risk of death. A modified Kaplan-Meier estimator with age as a time scale was used to calculate the lifetime risk of AF and associated 95% confidence intervals (CI) because the standard Kaplan-Meier estimator does not account for the competing risk of death and might thus over-estimate the absolute risk of AF [13, 31], that is, a participant who dies without AF can no longer be at risk of AF and death should be treated as a true-competing event.

Conceptually, the risk set at any age *j* contained all Rj subjects who were age *j* at some point during their follow-up. Subjects with new onset AF, died, or were censored at age *j* were removed from the risk sets for ages *j* + 1 and older, whereas subjects who were age *j* + 1 at entry were added to the risk set for age *j* + 1 [31]. The lifetime risk of AF with 95% CI was calculated using a SAS macro from the Framingham study [32]. The lifetime risk analysis is valuable in cross-cohort comparisons because it estimates the probability of being morbid by the disease at a specified attained age and would not be influenced by different follow-up duration across cohorts. Because few participants were followed beyond age 85 years in the UK Biobank, the lifetime risk of AF was calculated only through to attained age of 85 years.

We computed the lifetime risk of AF in subgroups of participants for each modifiable lifestyle factor separately and for the combinations. The lifetime risk estimates for the non-adverse or adverse lifestyle factor groups in UK Biobank were compared with those in the K-NHIS-HealS by a *z* ratio test (the difference in the lifetime risk between two groups divided by its standard error) to assess race/ethnic differences. In addition, we broke the analyses down further according to the number of adverse lifestyle factors and repeated the same cross-cohort comparisons.

Fine-Gray subdistribution hazard models [33] were used to compare the relative risk of AF for each modifiable lifestyle factor separately and for the number of adverse lifestyle factors, adjusting for the competing risk of death. The proportional hazard assumption was tested using Schoenfeld residuals [34].

All analyses were performed using SAS version 9.4 for Windows (SAS Institute) and R statistics version 4.0.2 (R Foundation for Statistical Computing), and a two-sided *p*-value < 0.05 was considered statistically significant.

### Sensitivity analysis

Several sensitivity analyses were performed in this study. First, we estimated the lifetime risk of AF using a more precise definition for alcohol and physical activity strata, total amount of alcohol consumed per week, and total amount of moderate-to-vigorous intensity physical activity (MVPA) per week. Second, we estimated the lifetime risk of AF among Asians in the UK Biobank (who were excluded from the main analysis) and compared it with East Asians in the K-NHIS-HealS to provide further insight on the role of race/ethnicity and modifiable lifestyle factors in incident AF. Further details and rationale for the sensitivity analyses are provided in Additional file 1: Supplementary Methods.

## Results

### Baseline characteristics by lifestyle risk factor burden

All the participants in the K-NHIS-HealS were East Asians, and 7660 incident AF occurred during a median of 7.2 years (5.9–7.8). Participants included from the UK Biobank were all White Europeans, and 15,723 incident AF occurred during a median of 11.8 years (11.0–12.5). Among East Asians and White Europeans, non-adverse (no adverse lifestyle factor) were present in 22.9% and 24.8% and adverse lifestyle factor profile (at least one adverse lifestyle factor) in 77.1% and 75.2%, respectively (Table 1).

**Table 1:** Baseline characteristics of the propensity score matched K-NHIS-HealS and UK Biobank participants

	**K-NHIS-HealS (*n* = 242,763)**	**UK Biobank (*n* = 242,763)**	***p*-value**	**SMD**
**Age, years**	57 (51–63)	57 (51–63)	1.000	< 0.001
**Female**	121,935 (50.2)	121,935 (50.2)	1.000	< 0.001
**Systolic BP, mmHg**	126 (115–136)	137 (125–150)	< 0.001	0.670
**Diastolic BP, mmHg**	80 (70–85)	82 (76–89)	< 0.001	0.384
**Blood pressure**				
Non-hypertensive	152,633 (62.9)	119,949 (49.4)	< 0.001	0.274
Hypertensive	90,130 (37.1)	122,814 (50.6)		
**Body mass index**				
Non-obese	235,452 (97.0)	184,839 (76.1)	< 0.001	0.642
Obese	7311 (3.0)	57,924 (23.9)		
**Smoking**				
Never or past smoker	201,434 (83.0)	217,317 (89.5)	< 0.001	0.191
Current smoker	41,329 (17.0)	25,446 (10.5)		
**Blood sugar**				
Non-diabetic	211,093 (87.0)	226,967 (93.5)	< 0.001	0.222
Diabetes	31,670 (13.0)	15,796 (6.5)		
**Alcohol**				
≤ 4 times per week	232,939 (96.0)	189,850 (78.2)	< 0.001	0.549
	9824 (4.0)	52,913 (21.8)		
**Physical activity**				
Non-sedentary	124,289 (51.2)	206,125 (84.9)	< 0.001	0.775
Sedentary	118,474 (48.8)	36,638 (15.1)		
**Lifestyle factor profiles**				
Non-adverse	55,500 (22.9)	60,325 (24.8)	< 0.001	0.047
Adverse	187,263 (77.1)	182,438 (75.2)		

*BP*, blood pressure; *HTN*, hypertension; *K-NHIS-HealS*, Korea National Health Insurance Service-Health Screening; *SMD*, standardized mean difference; *UK*, United Kingdom. All participants in K-NHIS-HealS were East Asian, and all included participants in UK Biobank were White European

The most common adverse lifestyle factor was physical inactivity in East Asians (48.8%), whereas it was hypertension in White Europeans (50.6%). Hypertension, obesity, and frequent alcohol consumption were more common in White Europeans, whereas cigarette smoking, diabetes mellitus, and physical inactivity were more common in East Asians (Table 1). The baseline characteristics of the individuals excluded from K-NHIS-HealS and UK Biobank cohorts are presented in Additional file 1: Table S3.

### Overall lifetime risk of AF

The overall lifetime risk of AF was 15.4% (95% CI, 14.7 to 15.9%) among East Asians and 20.9% (95% CI, 19.6 to 21.9%) among White Europeans (*p* < 0.001) (Additional file 1: Fig. S2), and the lifetime risk of AF in men was higher than in women regardless of race/ethnicity. In the cross-cohort comparison, the lifetime risk of AF for both men and women were higher in White Europeans than in East Asians. The period 10-year interval risks are presented in Additional file 1: Table S4. Similar trends with the main analysis were observed.

### Multiple adverse or borderline risk factors and the lifetime risk of AF

**Fig. 1:**
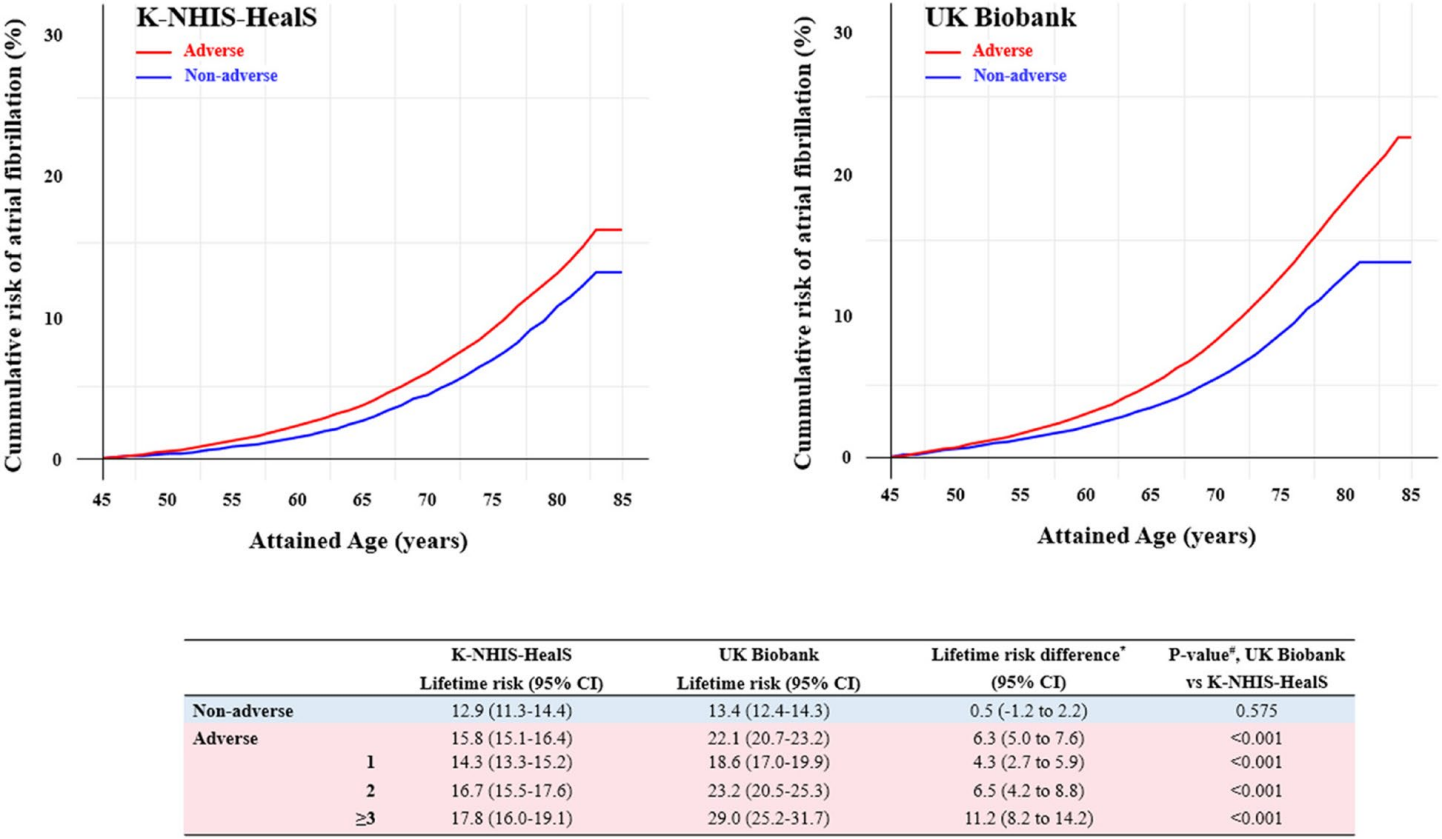
Lifetime risk of atrial fibrillation according to the modifiable lifestyle factor burden in K-NHIS-HealS and UK Biobank participants, after adjusting for the competing risk of death. CI, confidence interval. Other abbreviations are the same as Table 1. “*” symbol indicates the following: lifetime risk difference between White Europeans and East Asians and its associated 95% confidence intervals. “#” symbol indicates the following: comparison of the lifetime risk of subjects in the same lifestyle factor category between UK Biobank and K-NHIS-HealS participants using the Z-ratio test

Figure 1 shows the lifetime risk of AF by combination of modifiable lifestyle factors. In East Asians, the lifetime risk of AF was 12.9% (95% CI, 11.3 to 14.4%) in individuals with a non-adverse lifestyle factor profile and 15.8% (95% CI, 15.1 to 16.4) with an adverse lifestyle factor profile. In White Europeans, the lifetime risks of AF in individuals with non-adverse and adverse lifestyle factor profiles were 13.4% (95% CI, 12.4 to 14.3%) and 22.1% (95% CI, 20.7 to 23.2%), respectively. In the cross-cohort comparison, the lifetime risk of AF was similar between White Europeans and East Asians among those with non-adverse (*p* = 0.575) lifestyle factor profiles. In contrast, the lifetime risk of AF was significantly higher in White Europeans with an adverse lifestyle factor profile (*p* < 0.001). The period 10-year interval risks by modifiable lifestyle factor burden are presented in Additional file 1: Table S5, and the lifetime risk of AF by sex and modifiable lifestyle factor burden is presented in Additional file 1: Table S4 and Fig. S3. Similar trends with the main analysis were observed.

The lifetime risks of AF according to the number of adverse lifestyle factors in East Asians and White Europeans are presented in Fig. 1. The lifetime risk of AF was higher in White Europeans with any number of adverse lifestyle factors, and the lifetime risk difference between the two races incrementally increased with worsening adverse lifestyle factor burden. For example, the lifetime risk difference was 11.2% for ≥ 3 adverse lifestyle factors (17.8% East Asian vs 29.0% White European), whereas it was 4.3% for 1 adverse lifestyle factor (14.3% East Asian vs 18.6% White European).

**Fig. 2:**
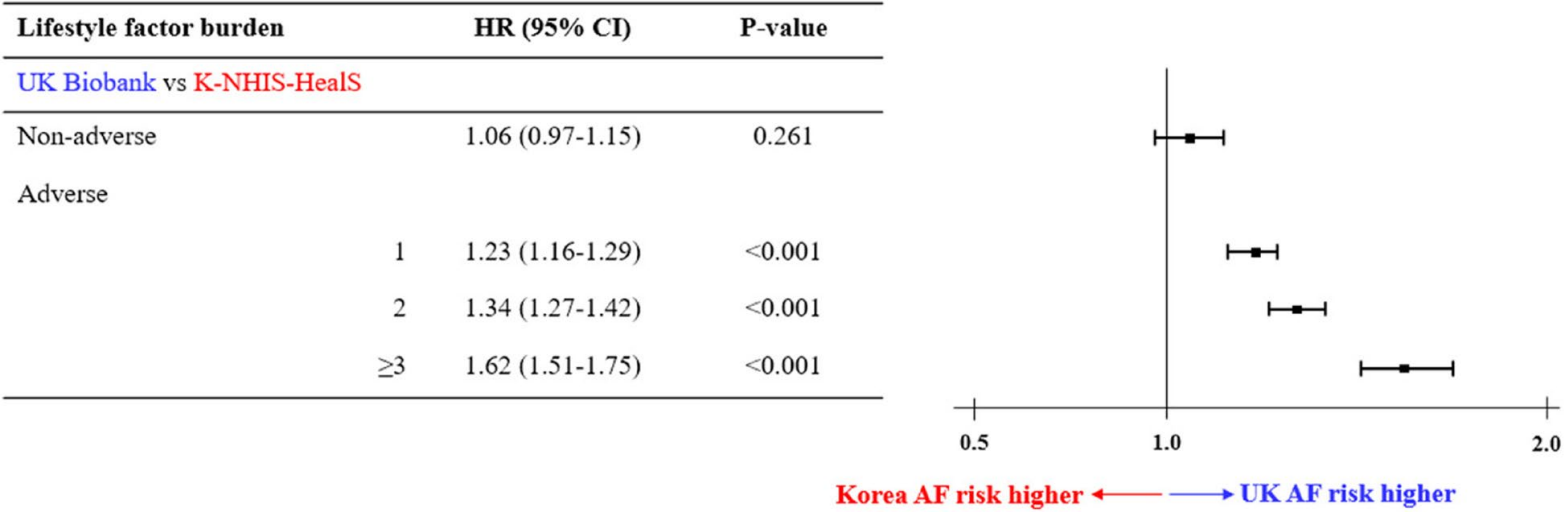
Number of adverse lifestyle factors and the relative risk of atrial fibrillation using Fine-Gray subdistribution hazard models across K-NHIS-HealS and UK Biobank participants, after adjusting for the competing risk of death. Abbreviations are the same as in Table 1

Figure 2 shows the relative risks of AF according to the number of adverse lifestyle factors across cohorts using Fine-Gray subdistribution hazard models, adjusting for the competing risk of death. Similar trends as in the lifetime risk analysis were identified. For example, the relative risk of AF was 62% higher (HR 1.62, 95% CI 1.51–1.75, *p* < 0.001) in White Europeans with ≥ 3 adverse lifestyle factors, whereas the relative risk of AF was similar between races among those with non-adverse lifestyle factor profiles (HR 1.06, 95% CI 0.97–1.15, *p* = 0.261). The relative risks of AF according to the number of adverse lifestyle factors within each cohort is presented in Additional file 1: Fig. S4.

### Single modifiable lifestyle factors and the lifetime risk of AF

Table 2 and Additional file 1: Fig. S5 report the lifetime risk of AF associated with a single modifiable lifestyle factor. The associated lifetime risk of AF was lowest when the lifestyle factor profile was non-adverse and increased as the lifestyle factor profile changed to adverse levels regardless of race/ethnicity (except for the smoking and physical activity strata in East Asians). In the cross-cohort comparison, the lifetime risk of AF in White European was consistently higher than in East Asians regardless of the lifestyle factor type, and the cross-cohort difference in the lifetime risk of AF increased as lifestyle factor changed from non-adverse to adverse levels. The period 10-year interval risks according to single lifestyle factors are presented in Additional file 1: Table S7.

**Table 2:** Lifetime risk of atrial fibrillation according to single lifestyle factors in K-NHIS-HealS and UK Biobank participants

	**K-NHIS-HealS**		**UK Biobank**		***p*-value**^**#**^**, UK Biobank vs K-NHIS-HealS**
	Case/PYR^a^	Lifetime risk (95% CI)	Case/PYR	Lifetime risk (95% CI)	
**Blood pressure**					
Non-hypertensive	3550/1,083,058	13.1 (12.2–13.9)	5677/1,374,606	17.5 (15.2–19.4)	< 0.001
Hypertensive	4110/674,320	17.8 (16.8–18.5)	10,046/1,375,085	22.9 (21.4–24.1)	< 0.001
**Body mass index**					
Non-obese	7356/1,705,614	15.2 (14.5–15.8)	10,137/2,106,716	18.5 (17.0–19.7)	< 0.001
Obese	304/51,763	20.4 (15.7–24.4)	5586/642,976	28.2 (25.8–30.0)	< 0.001
**Smoking**					
Never or past smoker	6360/1,475,598	15.4 (14.7–16.0)	13,970/2,467,108	20.6 (19.4–21.7)	< 0.001
Current smoker	1300/281,779	15.0 (13.1–16.2)	1753/282,583	23.4 (14.8–28.6)	0.002
**Blood sugar**					
Non-diabetic	6195/1,527,715	15.0 (14.3–15.7)	13,839/2,580,156	20.3 (18.9–21.4)	< 0.001
Diabetes	1465/229,663	16.9 (15.3–18.0)	1884/169,536	27.5 (25.7–28.9)	< 0.001
**Alcohol**					
≤4 times per week	7234/168,7150	15.3 (14.6–15.8)	11,875/2,154,283	19.9 (19.0–20.7)	< 0.001
	426/70,227	16.3 (13.3–18.4)	3848/595,408	23.6 (19.4–26.9)	< 0.001
**Physical activity**					
Non-sedentary	3509/862,723	15.5 (14.4–16.4)	12,999/2,339,580	20.5 (19.2–21.6)	< 0.001
Sedentary	4151/894,654	15.3 (14.4–15.9)	2724/410,111	22.9 (19.2–25.7)	< 0.001

*CI*, confidence interval; *PYR*, person-years. Other abbreviations are the same as Table 1

^a^Case indicates the number of incident atrial fibrillation diagnoses

^**#**^Comparison of the lifetime risk of each lifestyle factor category between the UK Biobank and K-NHIS-HealS cohorts using the Z-ratio test

**Fig. 3:**
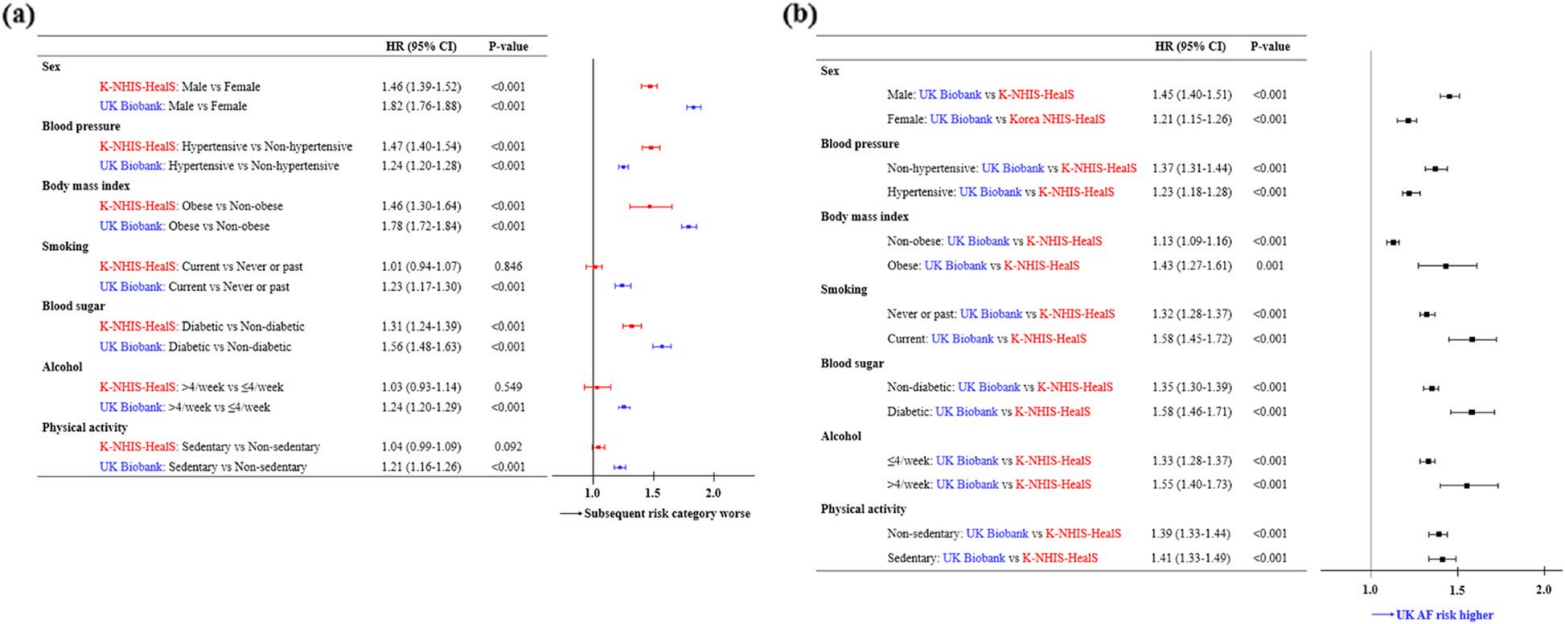
Single modifiable lifestyle factors and the relative risk of atrial fibrillation using Fine-Gray subdistribution hazard models **a** within and **b** across the K-NHIS-HealS and UK Biobank cohorts, after adjusting for the competing risk of death. Abbreviations are the same as in Table 1

The association between single lifestyle factor and the relative risk of AF using the Fine-Gray subdistribution hazard models within and across races are presented in Fig. 3. The relative risk of AF increased in White Europeans relative to East Asians as lifestyle factor changed from non-adverse to adverse levels (except for hypertension) (Fig. 3b).

### Sensitivity analysis

The lifetime risks of AF according to the amount of alcohol consumed per week and total amount of MVPA per week are presented in Additional file 1: Table S8. Heavy alcohol consumption (> 210 g/week) was associated with an increased lifetime risk of AF and sufficient physical activity (> 150 min/week of MVPA) was associated with a decreased lifetime risk of AF.

The lifetime risks of AF among Asians in UK Biobank compared with East Asians in K-NHIS-HealS are provided in Additional file 1: Table S9. Comparable lifetime risk of AF was observed (non-adverse, *p* = 0.521; adverse, *p* = 0.751).

## Discussion

### Principal findings

Using population-based, longitudinal data from East Asia and Europe, we estimated the lifetime risk of AF according to modifiable lifestyle factor profiles and compared the race/ethnic similarities and disparities. First, the overall lifetime risk of AF was higher in White Europeans than East Asians (20.9% White European vs 15.4% East Asian, *p* < 0.001). Second, the lifetime risk of AF was similar between the two races among individuals with healthy lifestyle factor profiles (13.4% White European vs 12.9% East Asian, *p* = 0.575). Third, the lifetime risk of AF was higher in White Europeans than East Asians among individuals with adverse lifestyle factor profiles (22.1% White European vs 15.8% East Asian, *p* < 0.001), and these differences became more pronounced as the burden of adverse lifestyle factor worsened (lifetime risk difference; 1 adverse lifestyle factor 4.3% to ≥ 3 adverse lifestyle factor 11.2%).

These findings suggest that the overall higher lifetime risk of AF observed in White Europeans compared with East Asians might be attributable to adverse lifestyle factors, and adherence to healthy lifestyle factor profiles would significantly reduce the lifetime risk of AF to about 1 in 8 regardless of race/ethnicity. In addition, BP and BMI were the most important lifestyle factors for increased lifetime risk of AF in both races. Despite underestimated in previous reports [13, 35], uncontrolled blood sugar increased the lifetime risk of AF comparable to that of BP and BMI in White Europeans.

Indeed, the modifiable lifestyle factors have a crucial role in the lifetime risk of AF, reflect the holistic or integrated care approach used in current guidelines for prevention of AF, and provide insight into race/ethnic differences observed in global AF epidemiology.

### Overall lifetime risk of AF

To our knowledge, only 8 studies have assessed the lifetime risk of AF in the community: 4 from US [13, 15–17], 1 from the Netherlands [14], 1 from Northern Europe [18], 1 from China [20], and 1 from Taiwan [19]. In the Framingham Heart Study, the overall lifetime risk of AF was about 1 in 4 with the last follow-up in 1999 [17], and in a follow-up study from the same cohort, lifetime risk of AF was about 1 in 3 with the last follow-up in 2013 [13]. In the Rotterdam study, the lifetime risk of AF was about 1 in 4 with the last follow-up in 1999 [14]. The lower lifetime risk of AF of about 1 in 5 among White Europeans in this study might be attributable to lifetime risk of AF estimated at the attained age of 85 years compared with attained age of 95 years in previous reports (Additional file 1: Table S10) [14, 17, 18]. However, the mean life expectancy in UK is 80 to 85 years, and lifetime risk of AF estimated beyond attained age of 85 years would overestimate the true lifetime risk of AF observed in the community. Data on the lifetime risk of AF in Asia are more limited. In a cohort of Chinese adults, the lifetime risk of AF was reported to be 1 in 5, and it was reported to be 1 in 7 in a cohort of Taiwanese adults [19, 20]. The overall lifetime risk of AF of 1 in 6.5 for South Koreans found in this study is comparable with previous reports on lifetime risk of AF among East Asians.

The similarities and disparities in the overall lifetime risk of AF between the two races/ethnicities pose planning and budgeting of AF policies that require clear understanding in AF epidemiology because not only East Asians in Western regions but also Whites in Asian regions are fast growing races/ethnicities.

### Modifiable lifestyle factor profiles and the lifetime risk of AF

In this study, worsening lifestyle factor profiles, from non-adverse to adverse, produced a greater increase in the lifetime risk of AF among White Europeans than East Asians, and more than 75% of participants had adverse lifestyle factor profiles in both cohorts. In a similar context, worsening burden of adverse lifestyle factors was associated with incrementally higher relative risk of AF among White Europeans (Fig. 2). Though debatable, symptom perception, health care access, and inherent genetic difference may contribute to the observed racial variation in lifetime risk of AF [36]. However, the public health implications of this study suggest AF interventions require focusing on integrated lifestyle modifications, especially among White Europeans because many healthcare resources are concentrated on finding and managing rather than preventing AF [11]. In addition, integrated lifestyle modification to prevent AF in East Asians should not still be underestimated given the reported higher risk of AF-related complications among Asians when AF once develop [37].

### Single modifiable lifestyle factor and the lifetime risk of AF

Among the modifiable lifestyle factors, BP and BMI were the most prominent factors for an increased lifetime risk of AF in White Europeans. These findings are consistent with previous reports on population attributable fractions for developing AF [35, 38]. We expand these observations to East Asians and found that BP and BMI were consistently the most important factors in increased lifetime risk of AF. From a public health perspective, our results highlight the potential of reducing lifetime risk of AF through BP and BMI optimization, which might be achieved from simple behavioral changes, and also the collateral improvements in other AF risk factors such as physical inactivity and heavy alcohol consumption via lifestyle interventions [11].

Our results also emphasize on the importance of blood sugar on increased lifetime risk of AF. Although diabetes is a well-known risk factor for AF, its contribution in lifetime risk of AF has been underestimated [13, 35]. In this study, the contribution of diabetes in increased lifetime risk of AF is comparable to that of obesity among White Europeans. Similar with BP and BMI, blood sugar optimization is achievable through lifestyle interventions including dietary changes, and in a recent meta-analysis, thiazolidinedione reduced incident AF, which might aid in prevention of AF in the setting of diabetes [39].

### Strengths and limitations

This study has multiple strengths. First, we used large, population-based cohort study data with large number of events and included participants who lived in the similar calendar periods for comparison. Second, the definitions used for outcomes and covariates were the same, and the data acquisition processes were similar between the two cohorts, which both used primary care, in-hospital admission, and death registries. Third, propensity score matching was used to reduce bias from cross-cohort comparisons. Fourth, because of race/ethnic heterogeneity of UK Biobank participants, we included only White Europeans for the current analysis. Fifth, the novel study design provides unique information on which lifestyle factors should health resources be focused on depending on race/ethnicity. For example, although policy makers should use health resources to control all domains of lifestyle factors, East Asians require more focus on BP and BMI, whereas White Europeans focus not only on BP and BMI but also equally on blood sugar to reduce the lifetime risk of AF.

This study also has several limitations. First, despite efforts to reduce bias from comparing two different cohorts from distant regions, concerns remain such as different health care access or hospitalization patterns. However, a comparable lifetime risk of AF among East Asians in K-NHIS-HealS and Asians in UK Biobank might mitigate those concerns. Second, although the underlying conditions were established from diagnosis (rather than self-reported), potential errors from over or underdiagnosis still remain, and race/ethnic difference in symptom perception might have contributed to this. Third, East Asian and White Europeans included from the K-NHIS-HealS and UK Biobank cohorts may not fully represent the respective race/ethnicity groups. For example, K-NHIS-HealS uses 10% random sampling of the national health claims, and UK Biobank includes healthy volunteers. Especially, healthy volunteers in the UK Biobank might have resulted in a lower lifetime risk of AF among White Europeans. Fourth, the lifestyle factor profiles were not assessed with time-dependent updates and might have changed during follow-up. Fifth, because many unmatched individuals were dropped from our analysis, the effects of modifiable lifestyle factors on lifetime risk of AF might have been underestimated. Sixth, longevity or severity of the adverse lifestyle factors was not considered in this study. In addition, other potential lifestyle factors such as dietary patterns, sleep disorders, and caffeine intake as well as non-lifestyle factors such as income, education, or genetic risks that may contribute to race/ethnic differences in the lifetime risk of AF were not controlled.

## Conclusions

In conclusion, the overall higher lifetime risk of AF in White Europeans compared with East Asians might be attributable to adverse lifestyle factors. Adherence to healthy lifestyle factors was associated with the lifetime risk of AF of about 1 in 8 regardless of race/ethnicity.

### Supplementary Information

**Additional file 1:** Supplementary Methods. **Table S1**. Definitions used to define modifiable lifestyle factors in both cohorts. **Table S2.** Definitions used to define (a) comorbidities and (b) outcomes in both cohorts. **Table S3.** Baseline characteristics of the excluded individuals. **Table S4.** Period 10-year interval risks of atrial fibrillation. **Table S5.** Period 10-year interval risks of atrial fibrillation according to modifiable lifestyle factor burden. **Table S6.** Lifetime risk of atrial fibrillation according to modifiable lifestyle factor burden and sex. **Table S7.** Period 10-year interval risks of atrial fibrillation according to single lifestyle factor. **Table S8.** Sensitivity analysis of the lifetime risk of atrial fibrillation by alcohol and physical activity using precise definitions. **Table S9.** Lifetime risk of atrial fibrillation among Asian in UK Biobank. **Table S10.** Comparison of lifetime risks of atrial fibrillation from from different longitudinal cohorts based on European ancestry. **Fig. S1.** Study flow chart. **Fig. S2.** Overall lifetime risk of atrial fibrillation in both cohorts. **Fig. S3.** Lifetime risk of atrial fibrillation according to modifiable lifestyle factor burden and sex. **Fig. S4.** Number of borderline or adverse lifestyle factors and relative risk of atrial fibrillation using Fine-Gray subdistribution hazard models within each cohort. **Fig. S5.** Lifetime risk of atrial fibrillation according to single lifestyle factor.

**Additional file 2.**

## Data Availability

Restrictions apply to the availability of these data from the Korea NHIS-HealS and UK Biobank. All data and materials can be obtained upon direct application.

## Abbreviations

AF: Atrial fibrillation
AHA: American Heart Association
BMI: Body mass index
BP: Blood pressure
MVPA: Moderate to vigorous physical activity
NHIS-HealS: National Health Insurance Service-Health Screening
UK: United Kingdom
US: United States
