# Supplementary material for: Modifiable lifestyle factors and lifetime risk of atrial fibrillation: longitudinal data from the Korea NHIS-HealS and UK Biobank cohorts
Source: BMC Med. 2024 May 13;22:194. doi: 10.1186/s12916-024-03400-4 (PMC11089782; doi:10.1186/s12916-024-03400-4)
Supplement: Supplementary file 1 — Additional file 1: Supplementary Methods. Table S1. Definitions used to define modifiable lifestyle factors in both cohorts. Table S2. Definitions used to define (a) comorbidities and (b) outcomes in both cohorts. Table S3. Baseline characteristics of the excluded individuals. Table S4. Period 10-year interval risks of atrial fibrillation. Table S5. Period 10-year interval risks of atrial fibrillation according to modifiable lifestyle factor burden. Table S6. Lifetime risk of atrial fibrillation according to modifiable lifestyle factor burden and sex. Table S7. Period 10-year interval risks of atrial fibrillation according to single lifestyle factor. Table S8. Sensitivity analysis of the lifetime risk of atrial fibrillation by alcohol and physical activity using precise definitions. Table S9. Lifetime risk of atrial fibrillation among Asian in UK Biobank. Table S10. Comparison of lifetime risks of atrial fibrillation from from different longitudinal cohorts based on European ancestry. Fig. S1. Study flow chart. Fig. S2. Overall lifetime risk of atrial fibrillation in both cohorts. Fig. S3. Lifetime risk of atrial fibrillation according to modifiable lifestyle factor burden and sex. Fig. S4. Number of borderline or adverse lifestyle factors and relative risk of atrial fibrillation using Fine-Gray subdistribution hazard models within each cohort. Fig. S5. Lifetime risk of atrial fibrillation according to single lifestyle factor. [file 12916_2024_3400_MOESM1_ESM.docx]

**Modifiable lifestyle factors and lifetime risk of atrial fibrillation: Longitudinal data from the Korea NHIS-HealS and UK Biobank cohorts**

Additional file 1

**Supplementary Methods**

**Table S1.** Definitions used to define modifiable lifestyle factors in K-NHIS-HealS and UK Biobank cohorts.

**Table S2.** Definitions used to define (a) comorbidities and (b) outcomes in K-NHIS-HealS and UK Biobank cohorts.

**Table S3.** Baseline characteristics of the excluded versus included individuals from the K-NHIS-HealS and UK Biobank cohorts.

**Table S4.** Period 10-year interval risks of atrial fibrillation in K-NHIS-HealS and UK Biobank cohorts.

**Table S5.** Period 10-year interval risks of atrial fibrillation according to modifiable lifestyle factor burden in K-NHIS-HealS and UK Biobank data.

**Table S6.** Lifetime risk of atrial fibrillation according to modifiable lifestyle factor burden and sex in K-NHIS-HealS and UK Biobank data.

**Table S7.** Period 10-year interval risks of atrial fibrillation according to single lifestyle factor in K-NHIS-HealS and UK Biobank data.

**Table S8.** Sensitivity analysis of the lifetime risk of atrial fibrillation by alcohol and physical activity using precise definitions.

**Table S9.** Lifetime risk of atrial fibrillation among Asian in UK Biobank compared with East Asian in K-NHIS-HealS.

**Table S10.** Comparison of lifetime risks of atrial fibrillation from specified-index age to attained age of 85 years from different longitudinal cohorts based on European ancestry.

**Fig. S1.** Study flow chart.

**Fig. S2**. Overall lifetime risk of atrial fibrillation in K-NHIS-HealS and UK Biobank cohorts, after adjusting for the competing risk of death.

**Fig. S3.** Lifetime risk of atrial fibrillation according to modifiable lifestyle factor burden and sex in K-NHIS-HealS and UK Biobank data.

**Fig. S4.** Number of borderline or adverse lifestyle factors and relative risk of atrial fibrillation using Fine-Gray subdistribution hazard models within the K-NHIS-HealS and UK Biobank cohorts, after adjusting for the competing risk of death.

**Fig. S5.** Lifetime risk of atrial fibrillation according to single lifestyle factor in K-NHIS-HealS and UK Biobank data.

**Supplementary Methods**

*More precise definition of alcohol and physical activity*

In this study, alcohol was categorized according to frequency of consumption rather than total amount of alcohol consumed per week and physical activity was categorized according to participation in regular physical activity rather than the total amount of physical activity per week for the main analysis. We used those categories because of missing data and heterogenous alcohol and physical activity questionnaire between the two cohorts. Therefore, we performed a sensitivity analysis among those with available data by using a more precise definition for alcohol and physical activity strata. The lifetime risk of AF was estimated by categorizing the amount of alcohol consumed per week as mild (<105g/week), moderate (≥105 to <210 g/week), and heavy (≥210 g/week); amount of physical activity was categorized as sedentary (0 min/week of MVPA), insufficient (<150 min/week of MVPA), and sufficient (≥150 min/week of MVPA).

*Lifetime risk of Asians in the UK Biobank cohort*

Among individuals with non-White ethnicity in the UK Biobank (who were excluded from the main analysis), we identified 7,186 individuals with Asian ethnicity. We estimated the lifetime risk of AF among those individuals using the same outcome and covariate definitions and compared that with the lifetime risk of AF of East Asians in the K-NHIS-HealS. This sensitivity analysis was included as a means for assessing systematic difference in cross-cohort comparisons.

*Empirical testing of the calipers used for propensity score matching*

Although a caliper of 0.01was used for propensity score matching in the main analysis, we additionally tested multiple calipers from 0.01 through 0.05 using 0.005 intervals to examine whether empirical testing for multiple calipers would reduce the number of unmatched individuals without introducing significant confounding. The number of individuals matched between the two cohorts using calipers from 0.010 through 0.035 were the same. The number of individuals matched increased when we used calipers from 0.040 through 0.050 but a significant difference in age and sex between the K-NHIS-HealS and UK Biobank cohort was identified. Therefore, we retained a caliper of 0.010 for the analysis.

**Table S1.** Definitions used to define modifiable lifestyle factors in K-NHIS-HealS and UK Biobank cohorts.

| **Blood pressure** | | |
| --- | --- | --- |
| Non-adverse | Normal or prehypertension | SBP <140 mmHg and DBP <90mmHg and no treatment for hypertension |
| Adverse | Hypertension | SBP ≥140mmHg or DBP ≥90mmHg or treatment for hypertension |
| **Body mass index** | | |
| Non-adverse | Normal or overweight | < 30 mg/kg^2^ |
| Adverse | Obese | ≥30 mg/kg^2^ |
| **Smoking** | | |
| Non-adverse | Never or past smoker |  |
| Adverse | Current smoker |  |
| **Blood sugar** | | |
| Non-adverse | Normal or prediabetes | Fasting blood glucose <125 mg/dL and no treatment for diabetes |
| Adverse | Diabetes | Fasting blood glucose > 126 mg/dL or treatment for diabetes |
| **Alcohol** | | |
| Non-adverse | ≤4 times per week |  |
| Adverse | > 4 times per week |  |
| **Physical activity** | | |
| Non-adverse | Non-sedentary | ≥ 30 min of LPA or ≥ 30 min of MPA or ≥ 20 min of VPA per week |
| Adverse | Sedentary | Less physical activity than the criteria for non-sedentary |

BP, blood pressure; DBP, diastolic blood pressure; HTN, hypertension; LPA, low-intensity physical activity; MPA, moderate-intensity physical activity; NHIS-HealS, National Health Insurance Service-Health Screening; SBP, systolic blood pressure; UK, United Kingdom; VPA, vigorous-intensity physical activity.

**Table S2.** Definitions used to define (a) comorbidities and (b) outcomes in K-NHIS-HealS and UK Biobank cohorts.

1. Comorbidities

|  | **Korea NHIS-HealS** | | **UK Biobank** | |
| --- | --- | --- | --- | --- |
|  | **Definitions** | **Used codes or conditions** | **Definitions** | **Used codes or conditions** |
| Atrial fibrillation | Defined from diagnosis^*^ | ICD-10: I48 | Defined from diagnosis^*^ | ICD-10: I48 |
| Hypertension | Defined from diagnosis^*^ plus treatment | ICD-10: I10, I11, I12, I13, I15  Treatment: all kinds of blood pressure lowering medications | Defined from diagnosis^*^ plus treatment | ICD-10: I10, I11, I12, I13, I15  Treatment: all kinds of blood pressure lowering medications |
| Diabetes mellitus | Defined from diagnosis^*^ plus treatment | ICD-10: E10, E11, E12, E13, E14  Treatment: all kinds of oral antidiabetics and insulin | Defined from diagnosis^*^ plus treatment | ICD-10: E10, E11, E12, E13, E14  Treatment: all kinds of oral antidiabetics and insulin |

*To ensure accuracy, comorbidities were established based on more than one hospital-inpatient or two outpatient (= primary care in United Kingdom) records of ICD-10 codes in the database.

1. Outcomes

|  | **Korea NHIS-HealS** | | **UK Biobank** | |
| --- | --- | --- | --- | --- |
|  | **Definitions** | **Used codes or conditions** | **Definitions** | **Used codes or conditions** |
| New onset atrial fibrillation (AF) | Defined from diagnosis^*^ or related death without previous insurance claim for AF | ICD-10: I48 | Defined from diagnosis^*^ or related death without previous history of AF | ICD-10: I48 |
| All-cause death | Data related to death were confirmed at the National Population Register of the Korea National Statistical Office, where deaths are centrally registered based on the death certificate. | The cause of death was determined based on the ICD-10 code written on the death certificate. | Data related to death were confirmed by the death registries of United Kingdom | The cause of death was determined based on the ICD-10 code written on the death certificate. |

*To ensure accuracy, comorbidities were established based on more than one hospital-inpatient or two outpatient (= primary care in United Kingdom) records of ICD-10 codes in the database.

**Table S3.** Baseline characteristics of the excluded versus included individuals from the K-NHIS-HealS and UK Biobank cohorts.

1. K-NHIS-HealS

|  | **Excluded**  **(n=214746)** | **Included**  **(n=242763)** | **P-value** |
| --- | --- | --- | --- |
| **Age, years** | 50 (46-63) | 57 (51-63) | <0.001 |
| **Female** | 88423 (41.2) | 121935 (50.2) | <0.001 |
| **Systolic BP, mmHg** | 125 (114-135) | 126 (115-136) | <0.001 |
| **Diastolic BP, mmHg** | 80 (70-85) | 80 (70-85) | <0.001 |
| **Blood pressure** | | | |
| Non-hypertensive | 124185 (57.9) | 152633 (62.9) | 0.007 |
| Hypertensive | 90469 (42.1) | 90130 (37.1) |  |
| Missing data |  | 0 |  |
| **Body mass index** | | | |
| Non-obese | 209325 (97.6) | 235452 (97.0) | <0.001 |
| Obese | 5254 (2.4) | 7311 (3.0) |  |
| Missing data | 167 | 0 |  |
| **Smoking** | | | |
| Never or past smoker | 150989 (79.3) | 201434 (83.0) | <0.001 |
| Current smoker | 39356 (20.7) | 41329 (17.0) |  |
| Missing data | 24401 | 0 |  |
| **Blood sugar** | | | |
| Non-diabetic | 190127 (88.6) | 211093 (87.0) | <0.001 |
| Diabetes | 24367 (11.3) | 31670 (13.0) |  |
| Missing data | 252 | 0 |  |
| **Alcohol** | | | |
| ≤4 times per week | 102987 (50.8) | 232939 (96.0) | 0.001 |
| >4 times per week | 7940 (3.9) | 9824 (4.0) |  |
| Missing data | 8823 | 0 |  |
| **Physical activity** | | | |
| Non-sedentary | 102987 (50.8) | 124289 (51.2) | 0.004 |
| Sedentary | 99867 (49.2) | 118474 (48.8) |  |
| Missing data | 11892 | 0 |  |
| **Prevalent atrial fibrillation** | 5019 (2.3) | 0 (0.0) | <0.001 |

Abbreviations are the same as Table S1.

1. UK Biobank

|  | **Excluded**  **(n=259658)** | **Included**  **(n=242763)** | **P-value** |
| --- | --- | --- | --- |
| **Age, years** | 59 (50-65) | 57 (51-63) | <0.001 |
| **Female** | 151397 (58.3) | 121935 (50.2) | <0.001 |
| **Systolic BP, mmHg** | 136 (124-150) | 137 (125-150) | <0.001 |
| **Diastolic BP, mmHg** | 82 (75-89) | 82 (76-89) | <0.001 |
| **Blood pressure** | | | |
| Non-hypertensive | 115829 (44.8) | 119949 (49.4) | 0.007 |
| Hypertensive | 142846 (55.2) | 122814 (50.6) |  |
| Missing data | 983 | 0 |  |
| **Body mass index** | | | |
| Non-obese | 192253 (74.9) | 184839 (76.1) | <0.001 |
| Obese | 64310 (25.1) | 57924 (23.9) |  |
| Missing data | 3095 | 0 |  |
| **Smoking** | | | |
| Never or past smoker | 229193 (89.3) | 217317 (89.5) | <0.001 |
| Current smoker | 27517 (10.7) | 25446 (10.5) |  |
| Missing data | 2948 | 0 |  |
| **Blood sugar** | | | |
| Non-diabetic | 173420 (90.9) | 226967 (93.5) | <0.001 |
| Diabetes | 17308 (9.1) | 15796 (6.5) |  |
| Missing data | 68930 | 0 |  |
| **Alcohol** | | | |
| ≤4 times per week | 209314 (81.1) | 189850 (78.2) | <0.001 |
| >4 times per week | 48844 (18.9) | 52913 (21.8) |  |
| Missing data | 1500 | 0 |  |
| **Physical activity** | | | |
| Non-sedentary | 178680 (82.0) | 206125 (84.9) | <0.001 |
| Sedentary | 39260 (18.0) | 36638 (15.1) |  |
| Missing data | 41718 | 0 |  |
| **Prevalent atrial fibrillation** | 7224 (2.8) | 0 (0.0) | <0.001 |

Abbreviations are the same as Table S1.

**Table S4.** Period 10-year interval risks of atrial fibrillation in K-NHIS-HealS and UK Biobank cohorts.

|  | | **K-NHIS-HealS** | | | **UK Biobank** | | |
| --- | --- | --- | --- | --- | --- | --- | --- |
|  | | **10-year risk** | **20-year risk** | **30-year risk** | **10-year risk** | **20-year risk** | **30-year risk** |
| Overall | Case/PYR**^†^** | 552/397586 | 2460/1056566 | 6129/1633522 | 872/456781 | 4797/1582477 | 12777/2558136 |
|  | Residual risk | 0.9 (0.8-1.0) | 3.2 (3.1-3.4) | 8.5 (8.3-8.7) | 1.3 (1.2-1.4) | 4.4 (4.3-4.6) | 11.6 (11.4-11.8) |
| Men | Case/PYR | 331/182459 | 1471/517995 | 3634/801359 | 507/212065 | 3139/772864 | 8249/1258184 |
|  | Residual risk | 1.2 (1.1-1.3) | 3.9 (3.6-4.1) | 9.8 (9.5-10.1) | 1.6 (1.5-1.8) | 5.7 (5.5-5.9) | 14.6 (14.3-14.9( |
| Women | Case/PYR | 221/215128 | 989/538571 | 2495/832164 | 365/244716 | 1658/809612 | 4528/1299952 |
|  | Residual risk | 0.7 (0.6-0.8) | 2.6 (2.5-2.8) | 7.1 (6.8-7.4) | 1.1 (1.0-1.2) | 3.2 (3.0-3.4) | 8.5 (8.3-8.8) |
| P-value**^*^**, Men vs Women | | <0.001 | <0.001 | <0.001 | <0.001 | <0.001 | <0.001 |

PYR, person-years. Other abbreviations are the same as in Table 1.

†Case indicates the number of atrial fibrillation diagnoses; PYR, person-years.

*Comparison of the 10, 20, and 30-year risk between men and women within each cohort using the Z-ratio test.

**Table S5.** Period 10-year interval risks of atrial fibrillation according to modifiable lifestyle factor burden in K-NHIS-HealS and UK Biobank data.

|  | | **K-NHIS-HealS** | | | **UK Biobank** | | |
| --- | --- | --- | --- | --- | --- | --- | --- |
|  | | **10-year risk** | **20-year risk** | **30-year risk** | **10-year risk** | **20-year risk** | **30-year risk** |
| Non-adverse | Case/PYR^†^ | 116/118781 | 457/273584 | 927/368776 | 239/160957 | 989/475095 | 2021/666643 |
|  | Residual risk | 0.7 (0.5-0.8) | 2.5 (2.3-2.7) | 6.8 (6.3-7.3) | 1.1 (1.0-1.3) | 3.3 (3.1-3.6) | 8.5 (8.1-8.8) |
| Adverse | Case/PYR | 436/278805 | 2003/782983 | 5202/1264746 | 633/295824 | 3803/1107382 | 10756/1891493 |
|  | Residual risk | 1.0 (0.9-1.1) | 3.5 (3.3-3.6) | 8.9 (8.6-9.1) | 1.5 (1.3-1.6) | 4.9 (4.7-5.0) | 12.5 (12.2-12.7) |
| P-value, Adverse vs Non-adverse | | <0.001 | <0.001 | <0.001 | <0.001 | <0.001 | <0.001 |

PYR, person-years. Other abbreviations are the same as in Table 1.

†Case indicates the number of atrial fibrillation diagnoses.

* Comparison of the 10, 20, and 30-year risk between a subsequent risk category and the least hazardous subcategory within each cohort using the Z-ratio test

**Table S6.** Lifetime risk of atrial fibrillation according to modifiable lifestyle factor burden and sex in K-NHIS-HealS and UK Biobank data.

|  | **K-NHIS-HealS** | | | **UK Biobank** | | | **P-value^#^, UK biobank vs K-NHIS-HealS** |
| --- | --- | --- | --- | --- | --- | --- | --- |
|  | **Case/PYR**^†^ | **Lifetime risk (95% CI)** | **P**^*^ | **Case/PYR** | **Lifetime risk (95% CI)** | **P** |  |
| Men |  |  |  |  |  |  |  |
| Non-adverse | 603/164425 | 14.8 (12.6-16.7) | - | 1301/268554 | 19.5 (17.7-21.2) | - | <0.001 |
| Adverse | 3831/693804 | 17.0 (15.9-17.8) | 0.036 | 8743/1081057 | 26.2 (23.9-27.9) | <0.001 | <0.001 |
| Women |  |  |  |  |  |  |  |
| Non-adverse | 471/218647 | 10.9 (8.5-13.1) | - | 1059/428533 | 10.9 (9.8-11.9) | - | 1.000 |
| Adverse | 2755/680501 | 14.5 (13.6-15.3) | 0.003 | 4620/971547 | 17.4 (15.7-18.9) | <0.001 | <0.001 |

CI, confidence interval; PYR, person-years. Other abbreviations are the same as in Table 1.

†Case indicates the number of atrial fibrillation diagnoses; PYR, person-years

* Comparison of the lifetime risk between a subsequent risk category and the least hazardous subcategory in the K-NHIS-HealS cohort using the Z-ratio test

# Comparison of the lifetime risk associated with the risk categories between the UK Biobank and K-NHIS-HealS cohorts using the Z-ratio test.

**Table S7.** Period 10-year interval risks of atrial fibrillation according to single lifestyle factor in K-NHIS-HealS and UK Biobank data.

|  | **K-NHIS-HealS** | | | **UK Biobank** | | |
| --- | --- | --- | --- | --- | --- | --- |
|  | **10-year risk** | **20-year risk** | **30-year risk** | **10-year risk** | **20-year risk** | **30-year risk** |
| **Blood pressure** | | | | | | |
| Non-hypertensive | 0.8 (0.7-0.9) | 2.7 (2.6-2.9) | 7.2 (6.9-7.5) | 1.2 (1.1-1.3) | 3.8 (3.6-4.0) | 9.8 (9.6-10.1) |
| Hypertensive | 1.4 (1.2-1.6) | 4.3 (4.0-4.6) | 10.3 (9.9-10.7) | 1.6 (1.4-1.8) | 5.3 (5.0-5.5) | 13.1 (12.8-13.4) |
| P-value^*^, Hypertensive vs Non-hypertensive | <0.001 | <0.001 | <0.001 | <0.001 | <0.001 | <0.001 |
| **Body mass index** | | | | | | |
| Non-obese | 0.9 (0.8-1.0) | 3.2 (3.0-3.3) | 8.4 (8.2-8.6) | 1.2 (1.1-1.3) | 3.7 (3.5-3.8) | 9.9 (9.7-10.1) |
| Obese | 1.5 (0.9-2.2) | 5.0 (4.0-5.9) | 11.4 (10.0-12.7) | 2.0 (1.8-2.3) | 6.9 (6.6-7.3) | 16.9 (16.4-17.4) |
| P-value, Obese vs Non-obese | 0.096 | <0.001 | <0.001 | <0.001 | <0.001 | <0.001 |
| **Smoking** | | | | | | |
| Past or never | 0.8 (0.7-0.9) | 3.1 (3.0-3.2) | 8.3 (8.0-8.5) | 1.3 (1.2-1.4) | 4.3 (4.2-4.5) | 11.4 (11.2-11.6) |
| Current | 1.4 (1.1-1.6) | 3.8 (3.5-4.1) | 9.7 (9.1-10.3) | 1.8 (1.5-2.1) | 5.3 (4.9-5.8) | 13.4 (12.7-14.1) |
| P-value, Current vs Past or never | <0.001 | <0.001 | <0.001 | <0.001 | <0.001 | <0.001 |
| **Blood sugar** | | | | | | |
| Non-diabetic | 0.9 (0.8-1.0) | 3.1 (3.0-3.2) | 8.2 (8.0-8.4) | 1.3 (1.2-1.4) | 4.2 (4.1-4.4) | 11.1 (10.9-11.3) |
| Diabetic | 1.5 (1.0-1.9) | 4.3 (3.8-4.9) | 10.3 (9.6-10.9) | 2.9 (2.1-3.6) | 8.4 (7.5-9.3) | 18.1 (17.1-19.1) |
| P-value, Diabetes vs Non-diabetic | 0.004 | <0.001 | <0.001 | <0.001 | <0.001 | <0.001 |
| **Alcohol** | | | | | | |
| Non-drinker | 0.9 (0.8-1.0) | 3.2 (3.1-3.3) | 8.4 (8.2-8.6) | 1.3 (1.2-1.4) | 4.4 (4.3-4.6) | 11.5 (11.2-11.7) |
| >4 times per week | 1.1 (0.4-1.8) | 4.0 (3.1-4.9) | 9.8 (8.6-10.9) | 1.2 (1.0-1.4) | 4.5 (4.2-4.8) | 12.1 (11.7-12.6) |
| P-value, > 4/week vs ≤4/week | 0.579 | 0.083 | 0.014 | 0.381 | 0.587 | 0.029 |
| **Physical activity** | | | | | | |
| Non-sedentary | 0.9 (0.8-1.0) | 3.2 (3.0-3.3) | 8.4 (8.1-8.7) | 1.3 (1.2-1.5) | 4.3 (4.2-4.5) | 11.3 (11.1-11.6) |
| Sedentary | 0.9 (0.8-1.1) | 3.3 (3.1-3.5) | 8.5 (8.2-8.8) | 1.4 (1.1-1.6) | 4.9 (4.6-5.3) | 13.3 (12.7-13.8) |
| P-value, Sedentary vs Non-sedentary | 1.000 | 0.381 | 0.644 | 0.488 | 0.009 | <0.001 |

PYR, person-years. Other abbreviations are the same as Table 1.

*Comparison of the 10, 20, and 30-year risk between a subsequent risk category and the least hazardous risk category within each cohort using the Z-ratio test

**Table S8.** Sensitivity analysis of the lifetime risk of atrial fibrillation by alcohol and physical activity using precise definitions.

|  | **K-NHIS-HealS** | | | **UK Biobank** | | | **P-value^#^, UK Biobank vs K-NHIS-HealS** |
| --- | --- | --- | --- | --- | --- | --- | --- |
|  | **Case/PYR**^†^ | **Lifetime risk (95% CI)** | **P**^*^ | **Case/PYR** | **Lifetime risk (95% CI)** | **P** |  |
| **Alcohol** |  |  |  |  |  |  |  |
| Mild (<105g per week) |  | ‡ | - | 2649/652785 | 17.1 (13.8-20.4) | - | ‡ |
| Moderate (≥105 to <210g per week) |  | ‡ | ‡ | 540/120586 | 18.3 (14.8-21.7) | 0.620 | ‡ |
| Heavy (≥210g per week) |  | ‡ | ‡ | 483/99166 | 21.7 (18.0-24.5) | 0.037 | ‡ |
| **Physical activity** |  |  |  |  |  |  |  |
| Sufficient (≥150 min/week MVPA^+^) | 82/22405 | 11.0 (7.9-13.8) | - | 1545/317430 | 18.9 (17.1-20.4) | - | <0.001 |
| Insufficient (<150 min/week MVPA) | 116/28110 | 12.4 (9.5-15.1) | 0.481 | 7545/1350048 | 20.9 (19.0-22.4) | 0.065 | <0.001 |
| Sedentary | 258/59852 | 15.3 (12.9-17.5) | 0.018 | 3975/688436 | 22.4 (18.7-25.0) | 0.022 | <0.001 |

CI, confidence interval; MVPA, moderate-to-vigorous intensity physical activity. Other abbreviations are the same as in Table 1.

†Case indicates the number of incident atrial fibrillation diagnoses.

* Comparison of the lifetime risk between a subsequent lifestyle factor category and the least hazardous lifestyle factor category in the K-NHIS-HealS cohort using the Z-ratio test.

# Comparison of the lifetime risk associated with each lifestyle factor category between the UK Biobank and K-NHIS-HealS cohorts using the Z-ratio test.

‡Cells without values had too few observations for lifetime risk estimation.

+ Time spent engaging in vigorous intensity physical activity was multiplied by 2 to calculate the total time spent engaging in MVPA.

**Table S9.** Lifetime risk of atrial fibrillation among Asian in UK Biobank compared with East Asian in K-NHIS-HealS.

CI, confidence interval; PYR, person-years. Other abbreviations are the same as in Table 1.

‡Cells without values had too few observations available for risk estimation.

# Comparison of the lifetime risk associated with the risk categories between the UK Biobank and K-NHIS-HealS cohorts using the Z-ratio test.

|  | **K-NHIS-HealS** | | **UK Biobank Asian** | | **P-value^#^, UK Biobank vs K-NHIS-HealS** |
| --- | --- | --- | --- | --- | --- |
|  | Case/PYR^†^ | Lifetime risk (95% CI) | Case/PYR | Lifetime risk (95% CI) |  |
| Non-adverse | 1074/383073 | 12.9 (11.3-14.4) | 80/25075 | 11.5 (7.5-15.5) | 0.521 |
| Adverse | 6586/1374305 | 15.8 (15.1-16.4) | 314/73747 | 15.4 (12.7-17.8) | 0.751 |

**Table S10.** Comparison of lifetime risks of atrial fibrillation from specified-index age to attained age of 85 years from different longitudinal cohorts based on European ancestry.

|  | **Men** | | | | **Women** | | | |
| --- | --- | --- | --- | --- | --- | --- | --- | --- |
| **Age, years** | **UK** | **Netherland** | **US** | **Northern Europe** | **UK** | **Netherland** | **US** | **Northern Europe** |
|  | UK Biobank by Park et al. (Current study) | Rotterdam study by Heeringa et al. | Framingham Heart Study by **Lloyd-Jones et al.** | BiomarCaRE cohort by Magnussen et al. | UK Biobank by Park et al.  (Current study) | Rotterdam study by Heeringa et al. | Framingham Heart Study by **Lloyd-Jones et al.** | BiomarCaRE cohort by Magnussen et al. |
| **30** |  |  |  | ~23.3 (~30.8) |  |  |  | ~20.7 (~31.3) |
| **40** |  |  | ~22.2 (26.0)^*^ | ~23.1 (~30.6) |  |  | ~14.4 (23.0) | ~20.6 (~31.2) |
| **45** | 25.4 |  |  |  | 16.2 |  |  |  |
| **50** | 25.1 |  | ~21.5 (25.9) | ~22.0 (~29.5) | 15.9 |  | ~14.1 (23.2) | ~20.5 (31.2) |
| **55** | 24.5 | 20.1 (23.8) |  |  | 15.4 | 16.3 (22.2) |  |  |
| **60** | 23.5 | 19.6 (23.3) | ~21.4 (25.8) | ~19.6 (~27.1) | 14.9 | 16.4 (22.3) | ~14.3 (23.4) | ~19.7 (30.3) |
| **65** | 22.1 | 18.7 (22.7) |  |  | 14.0 | 19.1 (22.1) |  |  |
| **70** | 19.6 | 17.5 (21.9) | ~18.8 (24.3) | ~14.9 (~22.4) | 12.5 | 14.7 (21.1) | ~12.8 (23.0) | ~16.8 (~27.4) |
| **75** | 15.5 | 14.9 (20.2) |  |  | 9.7 | 11.2 (18.3) |  |  |
| **80** | 8.8 | 9.2 (16.1) | ~12.6 (22.7) | ~5.3 (~12.8) | 5.5 | 7.4 (15.3) | ~7.9 (21.6) | ~8.3 (~18.9) |
| **85** |  | - (11.8) |  |  |  | - (11.8) |  |  |

*Lifetime risk of atrial fibrillation from specified-index age to attained age of 85 years; values in the parenthesis indicate lifetime risk of atrial fibrillation estimated from specified index-age to attained age of >85 years in the Rotterdam study, 94 years in the Framingham heart study, and >95 years in the BiomarCaRE cohort. Estimated values at attained age of 85 years were provided by Heeringa et al.^14^ in the Rotterdam study. In contrast, Lloyd-Jones et al.^17^ in the Framingham Heart Study and Magnussen et al.^18^ in the BiomarCaRE cohort did not provide estimated values at attained age of 85 years, and therefore the values were inferred from the lifetime risk-age curves.

**Fig. S1.** Study flow chart.

**
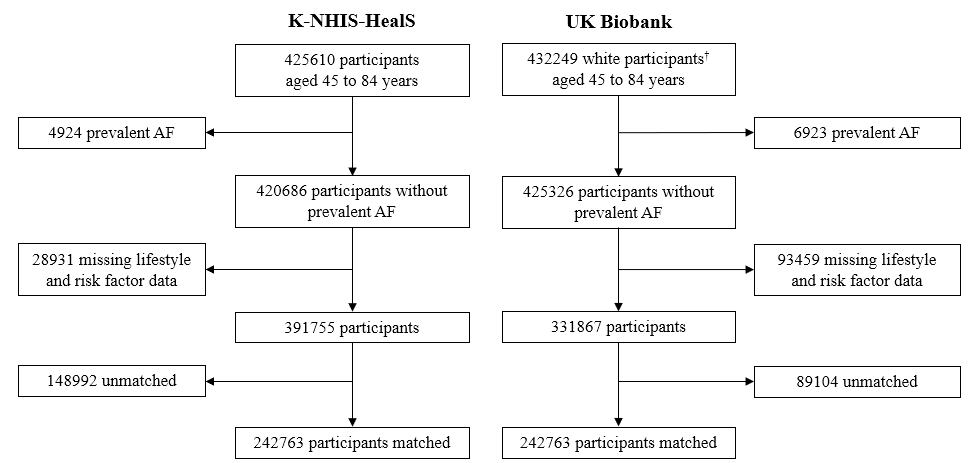
**

AF, atrial fibrillation. Other abbreviations are the same as in Table 1.

†Non-white race/ethnicity groups were excluded from UK Biobank.

**Fig. S2**. Overall lifetime risk of atrial fibrillation in K-NHIS-HealS and UK Biobank cohorts, after adjusting for the competing risk of death.


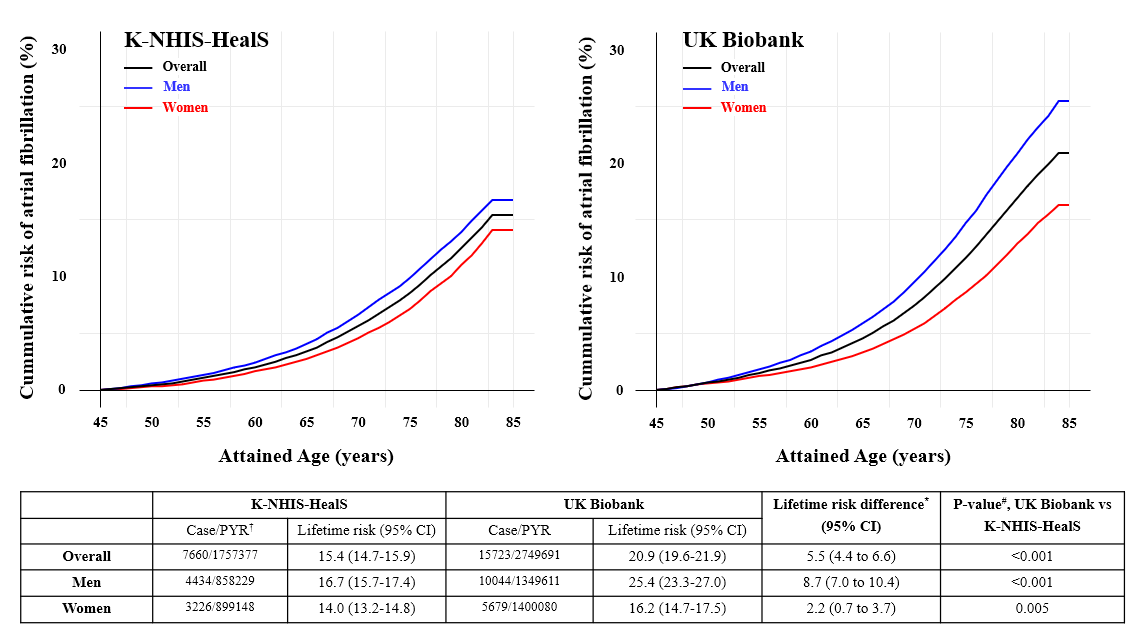


CI, confidence interval; PYR, person-years. Other abbreviations are the same as in Table 1.

†Case indicates the number of incident atrial fibrillation diagnoses.

*Lifetime risk difference between White Europeans and East Asians and its associated 95% confidence intervals.

#Comparison of the lifetime risk between UK Biobank with K-NHIS-HealS participants using Z-ratio test.

**Fig. S3.** Lifetime risk of atrial fibrillation according to modifiable lifestyle factor burden and sex in K-NHIS-HealS and UK Biobank data.


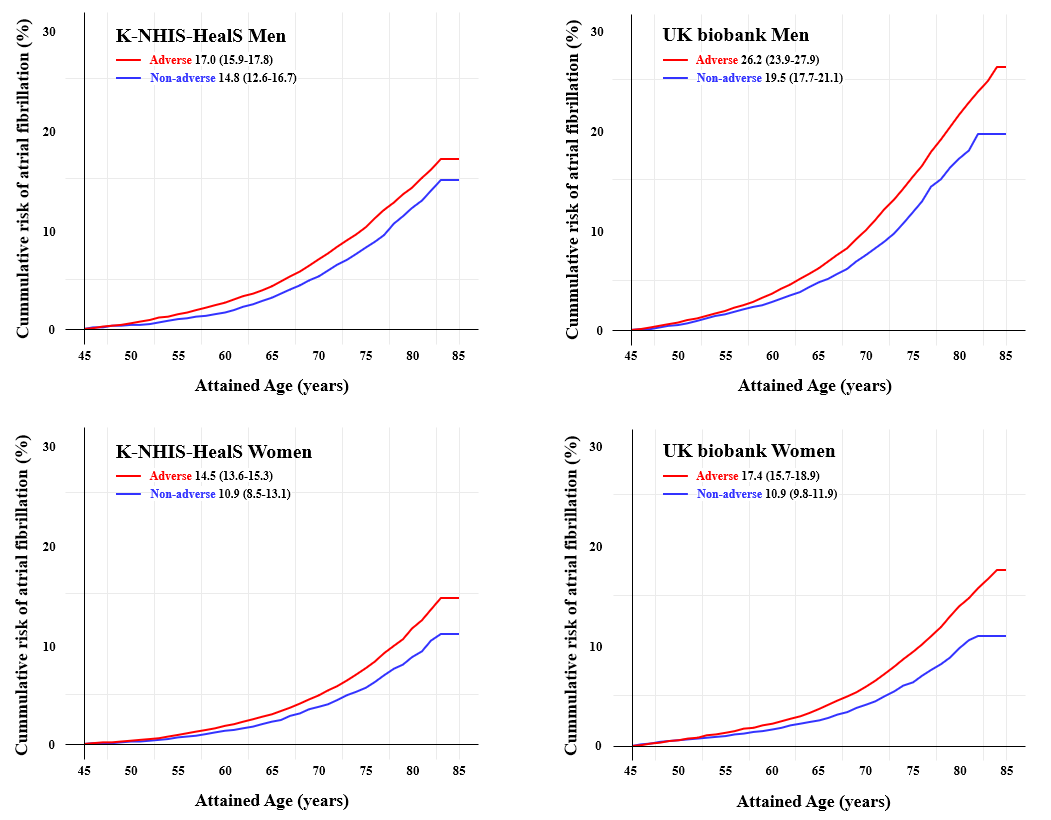


Abbreviations are the same as in Table 1.

**Fig. S4.** Number of borderline or adverse lifestyle factors and relative risk of atrial fibrillation using Fine-Gray subdistribution hazard models within the K-NHIS-HealS and UK Biobank cohorts, after adjusting for the competing risk of death.


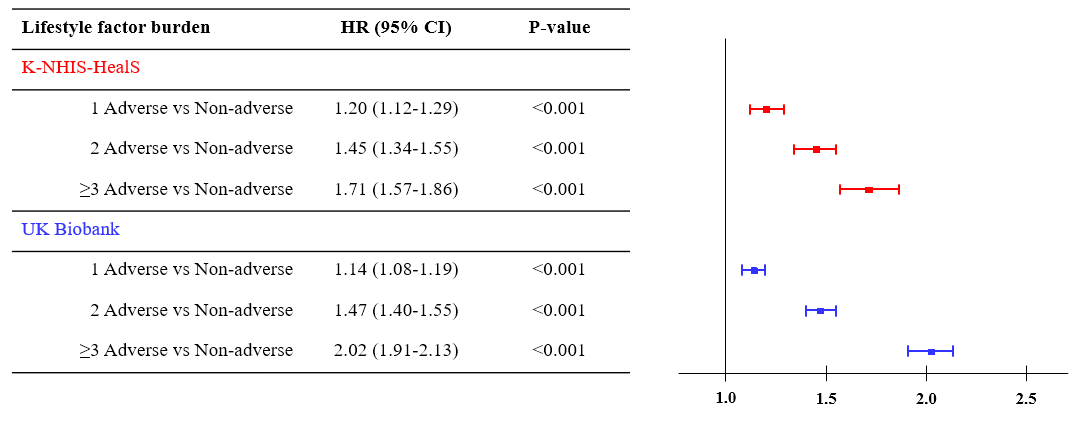


Abbreviations are the same as in Table 1.

**Fig. S5.** Lifetime risk of atrial fibrillation according to single lifestyle factor in K-NHIS-HealS and UK Biobank data.

**
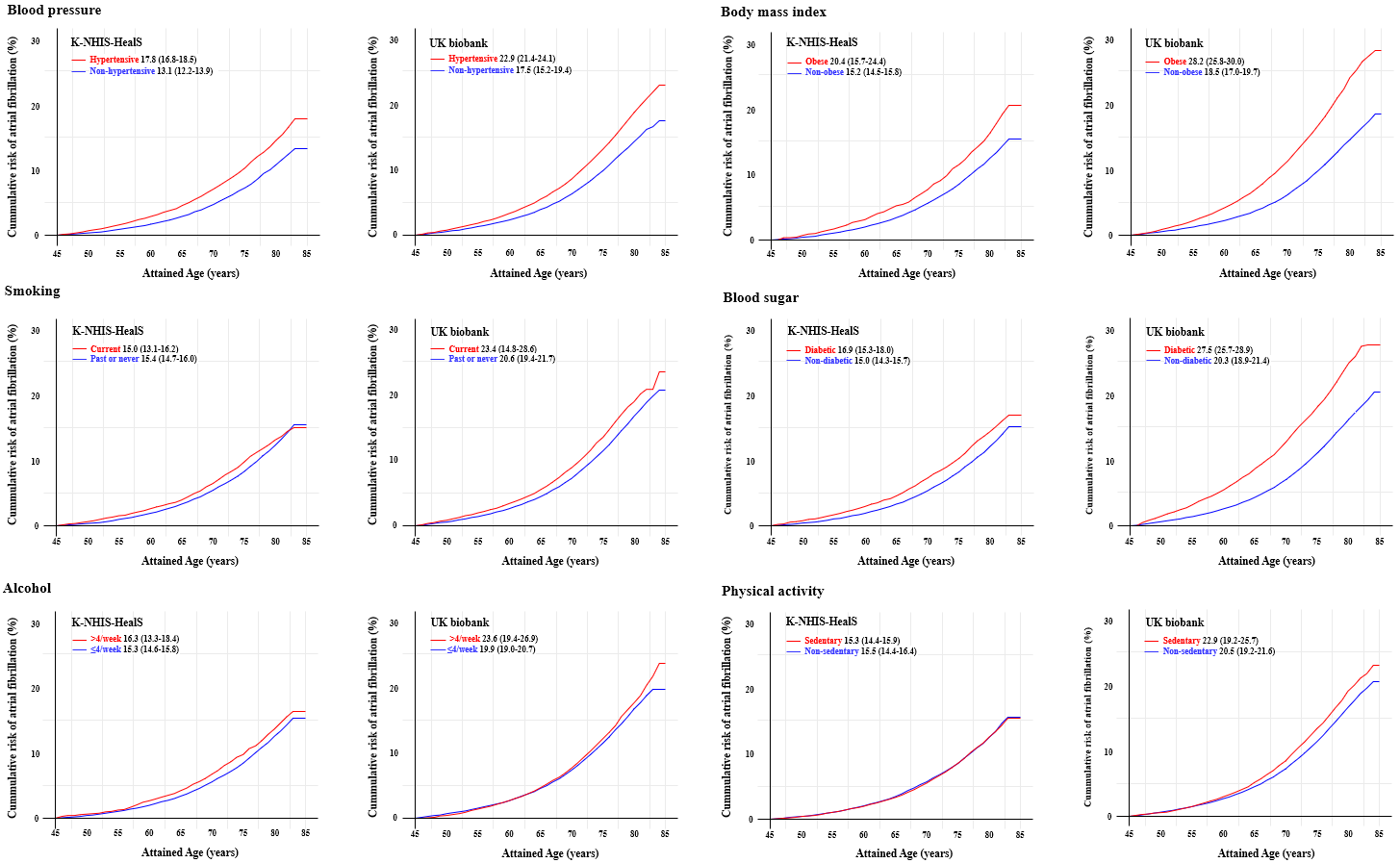
**

Abbreviations are the same as in Table 1.
