# Supplementary material for: Modifiable lifestyle factors and lifetime risk of atrial fibrillation: longitudinal data from the Korea NHIS-HealS and UK Biobank cohorts
Source: BMC Med. 2024 May 13;22:194. doi: 10.1186/s12916-024-03400-4 (PMC11089782; doi:10.1186/s12916-024-03400-4)
Supplement: Supplementary file 2 — Additional file 2. [file 12916_2024_3400_MOESM2_ESM.docx]

STROBE Statement—checklist of items that should be included in reports of observational studies

|  | Item No. | Recommendation | Page  No. | Relevant text from manuscript |
| --- | --- | --- | --- | --- |
| **Title and abstract** | 1 | (*a*) Indicate the study’s design with a commonly used term in the title or the abstract | 1 | **Modifiable lifestyle factors and lifetime risk of atrial fibrillation: Longitudinal data from the Korea NHIS-HealS and UK Biobank cohorts** |
|  |  | (*b*) Provide in the abstract an informative and balanced summary of what was done and what was found | 2 | **The overall higher lifetime risk of AF in White Europeans compared with East Asians might be attributable to adverse lifestyle factors. Adherence to healthy lifestyle factors was associated with a significantly lower lifetime risk of AF regardless of race/ethnicity.** |
| Introduction | | | |  |
| Background/rationale | 2 | Explain the scientific background and rationale for the investigation being reported | 3 | the reason for the higher incidence of AF among Europeans compared with Asians are yet to be fully understood |
| Objectives | 3 | State specific objectives, including any prespecified hypotheses | 3 | compared the lifetime risk of AF according to modifiable lifestyle factors in an East Asian cohort and assessed the differences with that from White Europeans |
| Methods | | | |  |
| Study design | 4 | Present key elements of study design early in the paper | 5-6 | Among the 391,755 and 331,867 eligible for analysis in the K-NHIS-HealS and UK Biobank, we carried out 1:1 propensity score matching using the nearest neighbor method with a caliper of 0.01. Variables used for propensity score matching was age and sex. After excluding unmatched pairs from both cohorts, 242,763 participants in each of the K-NHIS-HealS and UK Biobank cohorts consisted the final study population |
| Setting | 5 | Describe the setting, locations, and relevant dates, including periods of recruitment, exposure, follow-up, and data collection | 4-5 | Among the 514,764 participants in the K-NHIS-HealS, we included 425,610 individuals aged 45 to 84 years who participated in health screening examination between January 1, 2005 and December 31, 2010  Among the 502,421 individuals in the UK Biobank, we included individuals aged 45 to 84 years who participated in health screening examination between January 1, 2006 and December 31, 2010. |
| Participants | 6 | (*a*) *Cohort study*—Give the eligibility criteria, and the sources and methods of selection of participants. Describe methods of follow-up  *Case-control study*—Give the eligibility criteria, and the sources and methods of case ascertainment and control selection. Give the rationale for the choice of cases and controls  *Cross-sectional study*—Give the eligibility criteria, and the sources and methods of selection of participants | 4-5 | We excluded participants with prevalent AF (n=4,924); missing modifiable lifestyle factor data (n=28,931). A total of 391,755 individuals were eligible for the analysis.  We excluded participants with self-reported non-White race/ethnicity (n=24,567); prevalent AF (n=6,923); missing modifiable lifestyle factor data (n=93,459). A total of 331,867 individuals were eligible for the analysis. |
|  |  | (*b*) *Cohort study*—For matched studies, give matching criteria and number of exposed and unexposed  *Case-control study*—For matched studies, give matching criteria and the number of controls per case | 5-6 | Among the 391,755 and 331,867 eligible for analysis in the K-NHIS-HealS and UK Biobank, we carried out 1:1 propensity score matching using the nearest neighbor method with a caliper of 0.01. Variables used for propensity score matching was age and sex. After excluding unmatched pairs from both cohorts, 242,763 participants in each of the K-NHIS-HealS and UK Biobank cohorts consisted the final study population |
| Variables | 7 | Clearly define all outcomes, exposures, predictors, potential confounders, and effect modifiers. Give diagnostic criteria, if applicable | 5-6 | The modifiable lifestyle factors were selected based on the literature. The modifiable lifestyle factors included were blood pressure (BP), body mass index (BMI), cigarette smoking, diabetes, alcohol consumption, and physical activity. We defined non-adverse and adverse categories for each modifiable lifestyle factor as described in Table S1. Presence of multiple adverse lifestyle factor profiles were evaluated according to two levels: all lifestyle factors were non-adverse (non-adverse) or presence of any adverse lifestyle factor (adverse). |
| Data sources/ measurement | 8* | For each variable of interest, give sources of data and details of methods of assessment (measurement). Describe comparability of assessment methods if there is more than one group | *5-6* | The modifiable lifestyle factors were selected based on the literature. The modifiable lifestyle factors included were blood pressure (BP), body mass index (BMI), cigarette smoking, diabetes, alcohol consumption, and physical activity. We defined non-adverse and adverse categories for each modifiable lifestyle factor as described in Table S1. Presence of multiple adverse lifestyle factor profiles were evaluated according to two levels: all lifestyle factors were non-adverse (non-adverse) or presence of any adverse lifestyle factor (adverse). |
| Bias | 9 | Describe any efforts to address potential sources of bias | 7 | First, we estimated the lifetime risk of AF using a more precise definition for alcohol and physical activity strata; total amount of alcohol consumed per week and total amount of moderate-to-vigorous intensity physical activity (MVPA) per week. Second, we estimated the lifetime risk of AF among Asians in the UK Biobank (who were excluded from the main analysis) and compared it with East Asians in the K-NHIS-HealS to provide further insight on the role of race/ethnicity and modifiable lifestyle factors in incident AF. Further details and rationale for the sensitivity analyses are provided in Supplemental Methods. |
| Study size | 10 | Explain how the study size was arrived at | 5-6 | Among the 391,755 and 331,867 eligible for analysis in the K-NHIS-HealS and UK Biobank, we carried out 1:1 propensity score matching using the nearest neighbor method with a caliper of 0.01. Variables used for propensity score matching was age and sex. After excluding unmatched pairs from both cohorts, 242,763 participants in each of the K-NHIS-HealS and UK Biobank cohorts consisted the final study population (Figure S1). |

Continued on next page

| Quantitative variables | 11 | Explain how quantitative variables were handled in the analyses. If applicable, describe which groupings were chosen and why | 6, Additional file 1: Table S1 | Continuous variables are reported as medians (interquartile range) and compared using the Kruskal-Wallis test. Categorical variables are reported as numbers (percentages) and compared using the Chi-square or Fisher’s exact test. |
| --- | --- | --- | --- | --- |
| Statistical methods | 12 | (*a*) Describe all statistical methods, including those used to control for confounding | 6-7 | Continuous variables are reported as medians (interquartile range) and compared using the Kruskal-Wallis test. Categorical variables are reported as numbers (percentages) and compared using the Chi-square or Fisher’s exact test.We calculated the lifetime risk of AF from the index age of 45 years to the attained age of 85 years, accounting for the competing risk of death. A modified Kaplan-Meier estimator with age as a time scale was used to calculate the lifetime risk of AF and associated 95% confidence intervals because the standard Kaplan-Meier estimator does not account for the competing risk of death and might thus over-estimate the absolute risk of AF. That is, a participant who dies without AF can no longer be at risk of AF and death should be treated as a true-competing event.  Fine-Gray subdistribution hazard models were used to compare the relative risk of AF for each modifiable lifestyle factor separately and for the number of adverse lifestyle factors, adjusting for the competing risk of death. The proportional hazard assumption was tested using Schoenfeld residuals |
|  |  | (*b*) Describe any methods used to examine subgroups and interactions | 7-8 | First, we estimated the lifetime risk of AF using a more precise definition for alcohol and physical activity strata; total amount of alcohol consumed per week and total amount of moderate-to-vigorous intensity physical activity (MVPA) per week. Second, we estimated the lifetime risk of AF among Asians in the UK Biobank (who were excluded from the main analysis) and compared it with East Asians in the K-NHIS-HealS to provide further insight on the role of race/ethnicity and modifiable lifestyle factors in incident AF. Further details and rationale for the sensitivity analyses are provided in Supplemental Methods. |
|  |  | (*c*) Explain how missing data were addressed | 4 | We excluded participants with prevalent AF (n=4,924); missing modifiable lifestyle factor data (n=28,931).  We excluded participants with self-reported non-White race/ethnicity (n=24,567); prevalent AF (n=6,923); missing modifiable lifestyle factor data (n=93,459) |
|  |  | (*d*) *Cohort study*—If applicable, explain how loss to follow-up was addressed  *Case-control study*—If applicable, explain how matching of cases and controls was addressed  *Cross-sectional study*—If applicable, describe analytical methods taking account of sampling strategy |  |  |
|  |  | (*e*) Describe any sensitivity analyses | 7 | Several sensitivity analyses were performed in this study. First, we estimated the lifetime risk of AF using a more precise definition for alcohol and physical activity strata; total amount of alcohol consumed per week and total amount of moderate-to-vigorous intensity physical activity (MVPA) per week. Second, we estimated the lifetime risk of AF among Asians in the UK Biobank (who were excluded from the main analysis) and compared it with East Asians in the K-NHIS-HealS to provide further insight on the role of race/ethnicity and modifiable lifestyle factors in incident AF. Further details and rationale for the sensitivity analyses are provided in Supplemental Methods. |
| Results | | | | |
| Participants | 13* | (a) Report numbers of individuals at each stage of study—eg numbers potentially eligible, examined for eligibility, confirmed eligible, included in the study, completing follow-up, and analysed | 5-6 | After excluding unmatched pairs from both cohorts, 242,763 participants in each of the K-NHIS-HealS and UK Biobank cohorts consisted the final study population (Figure S1). |
|  |  | (b) Give reasons for non-participation at each stage | 4-5 | Additional file 1: Fig. S1 |
|  |  | (c) Consider use of a flow diagram | Additional file 1: Fig. S1 | Additional file 1: Fig. S1 |
| Descriptive data | 14* | (a) Give characteristics of study participants (eg demographic, clinical, social) and information on exposures and potential confounders | 7-8 | All the participants in the K-NHIS-HealS were East Asians, and 7,660 incident AF occurred during a median of 7.2 years (interquartile range, 5.9-7.8). Participants included from the UK Biobank were all White Europeans, and 15,723 incident AF occurred during a median of 11.8 years (interquartile range, 11.0-12.5). Among East Asians and White Europeans, non-adverse (no adverse lifestyle factor) were present in 22.9% and 24.8% and adverse lifestyle factor profile (at least one adverse lifestyle factor) in 77.1% and 75.2%, respectively (**Table 1**). |
|  |  | (b) Indicate number of participants with missing data for each variable of interest | 4 | We excluded participants with prevalent AF (n=4,924); missing modifiable lifestyle factor data (n=28,931).  We excluded participants with self-reported non-White race/ethnicity (n=24,567); prevalent AF (n=6,923); missing modifiable lifestyle factor data (n=93,459) |
|  |  | (c) *Cohort study*—Summarise follow-up time (eg, average and total amount) | 7 | All the participants in the K-NHIS-HealS were East Asians, and 7,660 incident AF occurred during a median of 7.2 years (5.9-7.8). Participants included from the UK Biobank were all White Europeans, and 15,723 incident AF occurred during a median of 11.8 years (11.0-12.5). |
| Outcome data | 15* | *Cohort study*—Report numbers of outcome events or summary measures over time | *7-8* | All the participants in the K-NHIS-HealS were East Asians, and 7,660 incident AF occurred during a median of 7.2 years (interquartile range, 5.9-7.8). Participants included from the UK Biobank were all White Europeans, and 15,723 incident AF occurred during a median of 11.8 years (interquartile range, 11.0-12.5). Among East Asians and White Europeans, non-adverse (no adverse lifestyle factor) were present in 22.9% and 24.8% and adverse lifestyle factor profile (at least one adverse lifestyle factor) in 77.1% and 75.2%, respectively (**Table 1**). |
|  |  | *Case-control study—*Report numbers in each exposure category, or summary measures of exposure |  |  |
|  |  | *Cross-sectional study—*Report numbers of outcome events or summary measures |  |  |
| Main results | 16 | (*a*) Give unadjusted estimates and, if applicable, confounder-adjusted estimates and their precision (eg, 95% confidence interval). Make clear which confounders were adjusted for and why they were included | 8-90 | The overall lifetime risk of AF was 15.4% (95% CI, 14.7 to 15.9%) among East Asians and 20.9% (19.6 to 21.9%) among White Europeans (p <0.001) (Figure S2), and the lifetime risk of AF in men was higher than in women regardless of race/ethnicity. |
|  |  | (*b*) Report category boundaries when continuous variables were categorized | Additional file 1: Table S1 | Additional file 1: Table S1 |
|  |  | (*c*) If relevant, consider translating estimates of relative risk into absolute risk for a meaningful time period | 9-10 | Figure 2 shows the relative risks of AF according to the number of adverse lifestyle factors across cohorts using Fine-Gray subdistribution hazard models, adjusting for the competing risk of death. Similar trends as in the lifetime risk analysis were identified. For example, the relative risk of AF was 62% higher (HR 1.62, 95% CI 1.51-1.75, **p**<0.001) in White Europeans with ≥3 adverse lifestyle factors whereas the relative risk of AF was similar between races among those with non-adverse lifestyle factor profiles (HR 1.06, 95% CI 0.97-1.15, p=0.261). |

Continued on next page

| Other analyses | 17 | Report other analyses done—eg analyses of subgroups and interactions, and sensitivity analyses | 10 | The lifetime risks of AF according to the amount of alcohol consumed per week and total amount of MVPA per week are presented in Table S8. Heavy alcohol consumption (>210 g/week) was associated with an increased lifetime risk of AF and sufficient physical activity (>150 min/week of MVPA) was associated with a decreased lifetime risk of AF.  The lifetime risks of AF among Asians in UK Biobank compared with East Asians in K-NHIS-HealS are provided in Table S9. Comparable lifetime risk of AF was observed (non-adverse, p=0.521; adverse, p=0.751). |
| --- | --- | --- | --- | --- |
| Discussion | | | | |
| Key results | 18 | Summarise key results with reference to study objectives | 10 | We used population-based, longitudinal data from East Asia and Europe to estimate the lifetime risk of AF according to modifiable lifestyle factor profiles, and then we assessed the race/ethnic similarities and disparities. First, the overall lifetime risk of AF was higher in White Europeans than East Asians (20.9% White European vs 15.4% East Asian, p<0.001). Second, **the lifetime risk of AF was similar between the two races among individuals with healthy lifestyle factor profiles (13.4% White European vs 12.9% East Asian, p=0.575). Third, the lifetime risk of AF was higher in White Europeans than East Asians among individuals with adverse lifestyle factor profiles (22.1% White European vs 15.8% East Asian, p<0.001), and these differences became more pronounced as the burden of adverse lifestyle factor worsened** (lifetime risk difference; 1 adverse lifestyle factor 4.3% to ≥3 adverse lifestyle factor 11.2%). |
| Limitations | 19 | Discuss limitations of the study, taking into account sources of potential bias or imprecision. Discuss both direction and magnitude of any potential bias | 14 | First, despite efforts to reduce bias from comparing two different cohorts from distant regions, concerns remain such as different health care access or hospitalization patterns. However, a comparable lifetime risk of AF among Asians in UK Biobank and East Asians in K-NHIS-HealS might mitigate those concerns. |
| Interpretation | 20 | Give a cautious overall interpretation of results considering objectives, limitations, multiplicity of analyses, results from similar studies, and other relevant evidence | 11-12 | These findings suggest that the overall higher lifetime risk of AF observed in White Europeans compared with East Asians might be attributable to adverse lifestyle factors and adherence to healthy lifestyle factor profiles would significantly reduce the lifetime risk of AF to about 1 in 8 regardless of race/ethnicity. In addition, BP and BMI were the most important lifestyle factors for increased lifetime risk of AF in both races. Despite underestimated in previous reports, uncontrolled blood sugar was associated with increased lifetime risk of AF comparable to that of BP and BMI. Compared with East Asians, White Europeans were influenced by multiple domains of lifestyle factors, in which holistic lifestyle modification intervention should be emphasized.  Indeed, the modifiable lifestyle factors have a crucial role in the lifetime risk of AF, reflect the holistic or integrated care approach used in current guidelines for prevention of AF, and provide insight into race/ethnic differences observed in global AF epidemiology. |
| Generalisability | 21 | Discuss the generalisability (external validity) of the study results | 14 | UK Biobank consists of healthy volunteers and may not fully represent the general UK population. Specifically, this possibility might have resulted in a lower lifetime risk of AF among White Europeans. Third, the K-NHIS-HealS uses 10% random sampling of the national health claims database, and are not fully guaranteed to capture the underlying health risk factors to represent the general South Korean population.  East Asian and White Europeans included from the K-NHIS-HealS and UK Biobank cohorts may not fully represent the respective race/ethnicity groups. |
| Other information | |  | | |
| Funding | 22 | Give the source of funding and the role of the funders for the present study and, if applicable, for the original study on which the present article is based | 15 | This research was supported by a grant from the Patient-Centered Clinical Research Coordinating Center (PACEN) funded by the Ministry of Health & Welfare, Republic of Korea (grant number: HC19C0130). |

*Give information separately for cases and controls in case-control studies and, if applicable, for exposed and unexposed groups in cohort and cross-sectional studies.

**Note:** An Explanation and Elaboration article discusses each checklist item and gives methodological background and published examples of transparent reporting. The STROBE checklist is best used in conjunction with this article (freely available on the Web sites of PLoS Medicine at http://www.plosmedicine.org/, Annals of Internal Medicine at http://www.annals.org/, and Epidemiology at http://www.epidem.com/). Information on the STROBE Initiative is available at www.strobe-statement.org.
